# Determining Occupational Performance Issues in Women with Breast Cancer Referred to Treatment Centers of Hamadan, Iran

**DOI:** 10.31557/APJCP.2019.20.4.1113

**Published:** 2019

**Authors:** Farkhondeh Jamshidi, Nazila Akbarfahimi, Seyed Ali Hosseini, Arezoo Shayan, Asal Fazeli

**Affiliations:** 1 *Department of Occupational Therapy, *; 3 *Department of Clinical Psychology, University of Social Welfare and Rehabilitation Sciences, Tehran,*; 2 *Faculty of Nursing and Midwifery, Mother and Child Care Research Center, Hamadan University of Medical Sciences, Hamadan, Iran. *

**Keywords:** Breast cancer, occupational performance, women

## Abstract

**Objective:**

Women with breast cancer experience functional limitations at the time of diagnosis and after the initial treatment of cancer. Such limitations interfere with participation in self-care, work affairs, and leisure activities. The present study aimed to determine occupational performance priorities in women with breast cancer who had referred to treatment centers in Hamadan, Iran.

**Methods:**

In this cross-sectional, descriptive-analytical study, 102 women with breast cancer who had referred to treatment centers in Hamadan were selected through convenience sampling. The participants’ information was gathered using their medical records and a demographic information questionnaire. Then, they were interviewed using the Canadian Occupational Performance Measure (COPM) to determine their occupational performance issues. The gathered data were coded and analyzed using the SPSS statistical software, version 16.

**Results:**

The results indicated that out of the 22 defined codes for the patients’ selected activities, 45.8%, 30.8%, and 23.4% belonged to self-care, productivity, and leisure domains, respectively.

**Conclusion:**

Women with breast cancer experience various occupational performance issues due to disease complications and received treatments. In the present study, self-care comprised the occupational performance priority. Determining the clients’ intervention priorities, which is among the bases of occupational therapy interventions, can help women with breast cancer reach the desired quality of life.

## Introduction

Cancer is the main general health issue around the world and the second cause of death in the U.S. Breast cancer is the most common cancer among women. According to American Cancer Society (ACS), 266,120 new breast cancer cases were estimated in the U.S. in 2018, resulting in 40,920 deaths (Siegel et al., 2015). Based on the Iranian cancer registry report in 2014, 109,000 cancer cases were found in the 31 provinces of the country. Additionally, 154 per 100,000 new cases were found in 2014. Besides, breast cancer was the most common cancer (31.3 per 100,000 population) among women (Haddad, 2011). In Hamadan province, 2,350 cancer cases were found annually. Among this population, 1380 cases were male and the rest were female. Breast cancer was the most common cancer among women in this province. Indeed, 187 new breast cancer cases have been found in the province in the recent year. 

Breast cancer can be treated either locally or systematically (Haddad, 2011). Treatment through surgery, chemotherapy, hormone therapy, and radiotherapy can decrease the risk of local recurrence and metastasis and increase the overall survival. Yet, treatment of breast cancer is accompanied with a wide range of occupational disorders resulting from complications, such as swollen lymph nodes, pain syndromes, peripheral neuropathy, and limited range of motion (Rietman et al., 2004; Magaldi et al., 2005; Merchant et al., 2008; Bevilacqua et al., 2012). Given the fact that Occupational performance refers to the ability to perform those tasks that make it possible to carry out occupational roles in a satisfying manner appropriate for the individuals developmental stage, culture, and environment (Pendleton, 2006), Thus, most women with breast cancer who undergo treatment will experience functional limitations (Lee et al., 2005). These limitations interfere with patients’ daily activities and are called participation limits (Organization, 2001). Such limitations refer to problems faced by individuals in self-care, productivity, and leisure activities at home and in the society (Organization, 2001). Satisfaction with the ability to do one’s daily activities, which is representative of functional health, is one of the dimensions of quality of life (Cella et al., 2002). Not only participation limits affect quality of life, but they may also lead to sustainable limits in the long run (Ness et al., 2006). Recent investigations have concluded that reduced functional health predicted lower overall survival in treated women with breast cancer (Braithwaite et al., 2010; DiSipio et al., 2011). Satisfaction with the ability to carry out one’s daily activities affected both quality and quantity of life (Lyons et al., 2015). In this regard, younger survivors of breast cancer were at a higher risk of participation limits after treatment (Beckjord et al., 2014). A prior study on 2910 cancer survivors also revealed that younger women reported more emotional and physical worries, but were less probable to receive care after treatment (Beckjord et al., 2014). Similarly, another research indicated that younger survivors of breast cancer were at a higher risk of disability, which probably resulted from responsibilities requiring physical efforts, such as child care, wage employment, and homemaking (Mackenzie, 2014).

Understanding the breast cancer survivors’ occupational perspective is of great importance because it represents their quality of life (Palmadottir, 2010). In fact, breast cancer survivors normally evaluate their health and quality of life from the occupational viewpoint. They report health and satisfaction with life in case they can take part in important activities. In addition, they usually express their goals using occupation-based expressions. In this context, they intend to gain the ability to do particular activities that let them carry out significant roles (Hoggan, 2014; Newman, 2013). A previous study showed that breast cancer survivors felt alive, healthy, and strong because they had the ability to do real work affairs. However, some women suffered from limited ability to cooperate in such valuable deeds due to deficiencies and fatigue resulting from treatment of cancer (Vrkljan, 2001).

Considering the short recovery period and the patients’ best performance after breast surgeries, due attention should be paid to rehabilitation. Most studies performed on breast cancer rehabilitation have focused on the main deficits. Yet, attentions are being attracted to a wide concept of health dimensions; i.e., the International Classification of Functioning, Disability, and Health (ICF) proposed by World Health Organization (WHO, 2001).

Although many studies have revealed deficiencies in breast cancer patients’ upper extremities (Rietman et al., 2004; Gosselink et al., 2003), a limited number of studies have focused on participation limits. In order to compensate for the lack of evidence-based rehabilitation interventions for improving breast cancer survivors’ participation, researchers require reliable information about how breast cancer and its treatment affect rehabilitation goals and survivors’ participation in daily activities. Therefore, the present study aims to investigate occupational performance issues in women suffering from breast cancer referred to treatment centers in Hamadan, Iran.

## Materials and Methods

This cross-sectional, descriptive-analytical study was conducted on women with breast cancer who had undergone treatment and had referred to MRI, Assembly of Donors, and Imam clinics in Hamadan from January to June 2016. According to the previous studies (Loubani-Hawaita et al., 2016) and considering power = 0.80 and α = 0.05, 102 subjects were selected using convenience sampling. Based on other studies conducted on the issue and consultation with an oncologist, the inclusion criteria of the study were being diagnosed with stage I to III breast cancer, passage of at least three months from surgery, having been healthy, having undergone medical treatments (chemotherapy, radiotherapy, and hormone therapy), being expected to return to normal living but having unsatisfactory occupational needs, and being willing to participate in the study. The exclusion criteria were recurrence of cancer, intellectual and cognitive impairments, communication and expression problems, and unwillingness to participate in the study. It should be noted that age and educational restrictions were not considered in this study.

The study data were collected using a demographic information form and Canadian Occupational Performance Measure (COPM). COPM is a unique instrument used by occupational therapists to determine changes in their clients’ self-concept of occupational performance over time (Law et al., 1998). This measure was invented by Canadian Department of National Health and Welfare and Canadian Association of Occupational Therapy in a wide research in 1988. This was done based on a certain occupational therapy model and consisted of self-care, productivity, and leisure dimensions. COPM deals with clients’ roles and expectations. In this context, clients are involved in activities at the very beginning of occupational therapy. Their involvement in treatment processes increases little by little and can be used in all development stages and disability groups (Law et al., 1998). Many researchers have confirmed the validity and reliability of COPM (Carpenter et al., 2001; Cup et al., 2003; Law et al., 1994; Ripat et al., 2001). In addition, studies have proved that this measure could be used in research as well as clinical environments. Using this instrument, specialists could identify their clients’ occupational performance issues and, consequently, increase their satisfaction (Tryssenaar et al., 1999; Pollock et al., 1998). 

Generally, COPM is used to determine the clients’ occupational performance issues. This is done through five stages. In the first step, the therapist asks the clients to think about a day in their lives; what they need, want, or expect but are not able to do or are not satisfied with the way they are done. In the second step, the clients are asked to prioritize their daily activities on a 10-point Likert scale. In this stage, the clients rate their performance as well as their satisfaction with the performance. The problems identified using COPM can provide the basis for designing outcomes and determining intervention priorities. In the fourth step; i.e., reevaluation, the clients rescore their performance and satisfaction after an appropriate time period from the intervention. Finally, changes in performance and satisfaction are computed. Considering the present study objectives, the first three stages were carried out by the therapist.

In the study by Dehghan et al., (2015), the content validity of the Persian version of COPM was found to be 80.95+0.222. Besides, its repeatability was 0.84 in performance and 0.87 in satisfaction scores. It should be noted that this measure is used through semi-structures interviews, which takes only 20-30 minutes to be carried out by an experienced occupational therapist (Dehghan et al., 2015).

After getting the required permissions from the Research Vice-chancellor of Hamadan University of Medical Sciences, registering the study in the Ethics Committee of the University (code: IR.UMSHA.REC.1395.32), referring to the intended centers, and investigating the patients’ records, the study participants were selected. The participants were informed about the study objectives and procedures and were reassured about the confidentiality of their information. Then, they signed written informed consents for taking part in the study. At first, demographic information form was completed and COPM was explained to the patients. The data were collected within three months. Then, the data were analyzed using the SPSS statistical software, version 16.

## Results

This study aimed to determine occupational performance priorities in women aging 30-75 years who suffered from breast cancer and had referred to treatment centers of Hamadan, Iran. The participant included 102 women with breast cancer who took part in interviews using COPM. Accordingly, the women mentioned 107 activities as problematic dimensions. Then, the researchers coded the identified issues. In this way, 22 codes were determined for the identified activities. Based on these codes, the issues were classified into self-care, productivity, and leisure categories. It should be mentioned that the patients’ age and education level were noted in the demographic information form (Table 1).

Out of the 107 issues mentioned by the study participants, 45.8%, 30.8%, and 23.4% belonged to self-care, productivity, and leisure dimensions, respectively (Table 2).

**Figure 1:**
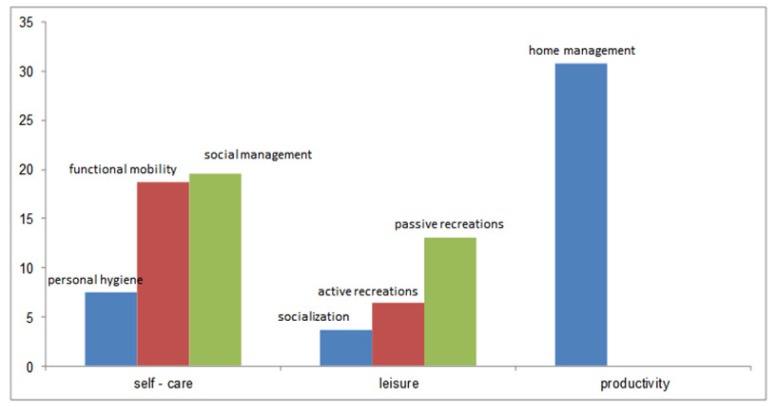
Frequency Distribution of Occupational Performance Issues in the Study Sample

Frequency distribution of the patients’ issues indicated that the highest frequency was related to self-care dimension. Out of the 49 issues identified in this dimension, 21 (19.6%), 20 (18.7%), and 8 issues (7.5%) were related to social management, functional mobility, and personal hygiene, respectively. Out of the 25 issues identified in leisure dimension, 14 (13.1%) were related to passive recreations, 7 (6.5%) were related to active recreations, and 4 (3.7%) were related to socialization. Moreover, all issues in the productivity dimension (n=33, 30.8%) belonged to home management (Table 3, Figure 1).

Most women’s problems were related to moving furniture (17, 15.9%) and washing clothes (15, 14%). On the other hand, the lowest frequencies were related to wearing clothes, washing dishes, eating, gardening, doing financial affairs, caring for personal hygiene (1, 0.9%), going out, exercising, and doing household chores (2, 1.9%) (Table 4).

## Discussion

The study results revealed that most breast cancer women’s occupational performance issues belonged to the self-care dimension. In this dimension, the highest frequency was related to social management (shopping), functional mobility (moving or carrying furniture), and personal hygiene (taking a shower and brushing hair). Productivity comprised the second priority and included home management (washing clothes). The lowest frequency was related to leisure dimension. In this dimension, the highest frequencies were related to passive recreations (doing art works), active recreations (studying), and socialization (visiting others).

Considering the fact that this was the first study on determination of occupational performance issues among women suffering from breast cancer in Iran, no similar studies have been published in this regard in the country. Therefore, international documents were explored to make comparisons. Izano et al., (2016) conducted a cohort study using a 3-month and a 12-month interview to assess physical problems among African-American women suffering from breast cancer. The results indicated that functional limitations after diagnosis of breast cancer included problems in mobility (walking half a mile) and upper extremities (pulling or pushing large objects, writing, or manipulating small objects) (Izano et al., 2016).

**Table 1:** The Demographic Information of the Women with Breast Cancer

variable / Characteristics	Frequency	Frequency (percent)
age		
30-39	13	12.74
40-49	35	34.31
50-59	37	36.27
60-69	13	12.74
70-79	4	3.92
total	102	100
Educational level		
Illiterate	27	26.47
Elementary	32	31.37
Guidance school	7	6.86
Diploma	30	29.41
Associate Degree	2	1.96
Bachelor	3	2.94
MA	1	0.98
Total	102	100

**Table 2:** Frequency Distribution of the Occupational Performance Issues in the Study Sampl

variable	Frequency	Frequency (percent)
Problems		
Self-care area	49	45.8
Productivity area	33	30.8
Leisure area	25	23.4
Total	107	100

**Table 3:** Frequency Distribution of the Occupational Performance Issues in the Study Sample

Variable			Frequency	Frequency (percent)	Percentage of area frequency
problems	Self-care area	Personal hygiene	8	7.5	16
		Functional mobility	20	18.7	41
		Social management	21	19.6	43
	leisure area	socialization	4	3.7	16
		Active recreations	7	6.5	28
		passive recreations	14	13.1	56
	Productivity area	Home management	33	30.8	100
		Total	107	100	Each area is 100

**Table 4:** Frequency Distribution of the Coded Issues in the Study Sample

Issues		Frequency	Percentage
1	Moving	17	15.9
2	Cooking	4	3.7
3	Shopping	12	11.2
4	Wearing clothes	1	0.9
5	Taking a shower	5	4.7
6	Cleaning the house	6	5.6
7	Going out	2	1.9
8	Visiting others	4	3.7
9	Studying	3	2.8
10	Caring for personal hygiene	1	0.9
11	Doing art works	13	12.1
12	Washing clothes	15	14
13	Being active	8	7.5
14	Exercising	2	1.9
15	Brushing hair	5	4.7
16	Doing financial affairs	1	0.9
17	Gardening	1	0.9
18	Going out of the house	3	2.8
19	Eating	1	0.9
20	Washing dishes	1	0.9
21	Household chores	2	1.9
Total frequency and frequency percentage		107	100

Ness et al., (2006), cited in Campbell et al., (2012), conducted a national health and nutrition survey that was a large population-based investigation of non-institutional adults in the U.S. to assess the prevalence of occupational limitations in cancer survivors in comparison to non-cancer controls. In that study, occupational limitations were evaluated using SF-36 based on some questions regarding participation limits and occupational scales. Accordingly, occupational limitations were rated as “small problem”, “large problem”, or “cannot be done” through self-report. Although that study involved all types of cancer, breast cancer was the most common one among women (25.4%). The results indicated that in comparison to the individuals without any history of cancer, recent cancer survivors (less than 5 years after diagnosis) and long-term ones (5 years) were more probable to report an occupational limitation. For instance, problem with walking 1.4 miles was reported by 8.7% of non-cancer individuals, 21.9% of recent cancer survivors, and 22.7% of long-term survivors. Additionally, 30.5% of recent cancer survivors and 31.3% of long-term ones compared to 13% of non-cancer controls reported participation limitations. These results implied that even after controlling for age, a considerable percentage of adult cancer survivors suffered from participation limits and functional deficits years after diagnosis. Thus, cancer survivors might benefit from rehabilitation services even years after treatment of their disease (Ness et al., 2006; Campbell et al., 2012). 

A systematic review conducted by Bijker et al., (2017) showed that occupational disorders could severely hinder breast cancer survivors from cooperation in activities. This would provide the ground for occupational health specialists to increase the opportunities for breast cancer survivors to return to work by taking their disease perceptions and work expectations into account (Bijker et al., 2017).

Karki et al., (2005) performed a prospective study to investigate participation limits in women with breast cancer using modified behavioral assessment scale. The results showed that lifting objects with upper extremities worsened the disorders in 59 patients (61.5%) in the 6-month follow-up and in 54 patients (56.3%) in the 12-month follow-up. Carrying with upper extremities also worsened the disorders in 51 patients (53.1%) in the 6-month follow-up and in 47 patients (49%) in the 12-month follow-up. These results were in line with those of the current research, which revealed that the highest frequency of women’s problems was related to moving and carrying furniture. The results also indicated that lifting one’s hand above one’s head worsened the disorders in 51 subjects (53.1%) in the 6-month follow-up and in 41 ones (42.7%) in the 12-month follow-up. Moreover, postoperative disorders interfered with sleeping in 37 (38.5%) and 34 (35.4%) participants in the 6- and 12-month follow-ups, respectively. However, patients experienced fewer problems in self-care. In this regard, only 10 patients (10.4%) had complaints or could not manage themselves in the 6-month follow-up and 8 ones (8.3%) had self-care limitations in the 12-month follow-up. Additionally, 16 patients (16.7%) in the 6-month follow-up and 15 ones (15.6%) in the 12-month follow-up experienced participation limits. Considering participation in homemaking, 31 patients (32.3%) had limitations in both 6- and 12-month follow-ups. Finally, 24 subjects (25%) in the 6-month follow-up and 16 ones (16.7%) in the 12-month follow-up experienced limitations in leisure activities (Karki et al., 2017).

Loubani-Hawaita et al., (2016) used COPM to identify women’s working priorities after treatment of breast cancer. According to the results, the five daily activities that received the highest scores included working (33%), homemaking (27%), shopping (20%), self-care (11%), and social activities/leisure (9%). Consistently, the present study findings demonstrated the least importance of leisure activities (Loubani-Hawaita et al., 2016).

Overall, investigation of the above-mentioned studies indicated that the priorities of occupational performance issues among women with breast cancer were different in various countries. Yet, determination of intervention priorities, which is among the bases of occupational therapy, could help such women reach the desired quality of life.

Since the present study participants were from both urban and rural areas, the results can be generalized to the whole region. Nonetheless, considering the cross-sectional design of the study and utilization of an available instrument (COPM), the results should be generalized with due caution. Hence, future cohort studies are recommended to be done using various quantitative scales and precise qualitative interviews in different parts of the country, so that Iranian occupational therapists would have access to occupational performance issues among women suffering from breast cancer.

In conclusion, the results of the current study showed that most breast cancer women’s priorities were related to the self-care dimension. In this dimension, the highest frequency belonged to moving and carrying furniture (functional mobility), washing clothes (home management), doing art works (passive leisure activities), and shopping (social management). Therefore, occupational therapists working in rehabilitation centers of Hamadan have to pay attention to the above-mentioned activities to plan interventions, analyze activities, and eventually improve patients’ quality of life.

## Statement conflict of Interest

In this study, there is no conflict of interest.

## References

[B1] Beckjord EB, Reynolds KA, Van Londen G (2014). Population-level trends in posttreatment cancer survivors concerns and associated receipt of care: results from the 2006 and 2010 LIVESTRONG surveys. J Psychosoc Oncol.

[B2] Bevilacqua JLB, Kattan MW, Changhong Y (2012). Nomograms for predicting the risk of arm lymphedema after axillary dissection in breast cancer. Ann Surg Oncol.

[B3] Bijker R, Duijts SF, Smith SN (2017). Functional impairments and work-related outcomes in breast cancer survivors: A systematic review. J Occup Rehabil.

[B4] Braithwaite D, Satariano WA, Sternfeld B (2010). Long-term prognostic role of functional limitations among women with breast cancer. J Natl Cancer Inst.

[B5] Campbell KL, Pusic AL, Zucker DS (2012). A prospective model of care for breast cancer rehabilitation: function. Cancer.

[B6] Carpenter L, Baker GA, Tyldesley B (2001). The use of the Canadian occupational performance measure as an outcome of a pain management program. Can J Occup Ther.

[B7] Cella D, Nowinski CJ (2002). Measuring quality of life in chronic illness: the functional assessment of chronic illness therapy measurement system. Arch Phys Med Rehabil.

[B8] Cup EH, Scholte op Reimer W, Thijssen MC, van Kuyk-Minis M (2003). Reliability and validity of the Canadian Occupational Performance Measure in stroke patients. Clin Rehabil.

[B9] Dehghan L, Dalvand H, Pourshahbaz A (2015). Translation of Canadian occupational performance measure and testing Persian version validity and reliability among Iranian mothers of children with cerebral palsy. JMR.

[B10] DiSipio T, Hayes S, Battistutta D, Newman B, Janda M (2011). Patterns, correlates, and prognostic significance of quality of life following breast cancer. Psychooncology.

[B11] Gosselink R, Rouffaer L, Vanhelden P (2003). Recovery of upper limb function after axillary dissection. J Surg Oncol.

[B12] Haddad CSF (2011). Radioterapia adjuvante no cancer de mama operavel. Femina.

[B13] Hoggan C (2014). Insights from breast cancer survivors: The interplay between context, epistemology, and change. Adult Educ Quart.

[B14] Izano M, Satariano WA, Hiatt RA, Braithwaite D (2016). The impact of functional limitations on long-term outcomes among African-American and white women with breast cancer: a cohort study. BMJ Open.

[B15] Karki A, Simonen R, Malkia E, Selfe J (2005). Impairments, activity limitations and participation restrictions 6 and 12 months after breast cancer operation. Journal of rehabilitation medicine: J Rehabil Med Suppl.

[B16] Law M, Baptiste S, Carswell A (1998). Canadian Occupational Performance Measure (COPM), CAOT Publications. ACE.

[B17] Law M, Polatajko H, Pollock N (1994). Pilot testing of the Canadian occupational performance measure: clinical and measurement issues. Can J Occup Ther.

[B18] Lee J, Dibble SL, Pickett M, Luce J (2005). Chemotherapy - induced nausea/vomiting and functional status in women treated for breast cancer. Cancer Nurs.

[B19] Loubani-Hawaita K, Schreuer N, Milman U (2016). Participation in daily activities among working women following breast cancer. OJTR.

[B20] Lyons KD, Svensborn IA, Kornblith AB, Hegel MT (2015). A content analysis of functional recovery strategies of breast cancer survivors. Otjr-Occup Part Heal.

[B21] Mackenzie CR (2014). It is hard for mums to put themselves first”: how mothers diagnosed with breast cancer manage the sociological boundaries between paid work, family and caring for the self. Soc Sci Med.

[B22] Magaldi CM, Barros ACS, Magaldi FM (2005). Avaliacao da morbidade e funcionalidade do membro superior em mulheres submetidas a linfadenectomia axilar total e biopsia de linfonodo sentinela por cancer de mama. Rev Bras Mastologia.

[B23] Merchant C, Chapman T, Kilbreath S, Refshauge K, Krupa K (2008). Decreased muscle strength following management of breast cancer. Disabil Rehabil.

[B24] Ness KK, Wall MM, Oakes JM, Robison LL, Gurney JG (2006). Physical performance limitations and participation restrictions among cancer survivors: a population-based study. Ann Epidemiol.

[B25] Newman RM (2013). Re-defining one’s occupational self 2 years after breast cancer: A case study. Work.

[B26] Organization WH (2001). International classification of functioning, disability and health.

[B27] Palmadottir G (2010). The role of occupational participation and environment among Icelandic women with breast cancer: A qualitative study. Scand J Occup Ther.

[B28] Pendleton H, Schultz-Krohn W (2006). Pedretti’s occupational therapy: practice skills for physical dysfunction.

[B29] Pollock N, Stewart D (1998). Occupational performance needs of school-aged children with physical disabilities in the community. Phys Occup Ther Pedl.

[B30] Rietman JS, Dijkstra PU, Debreczeni R (2004). Impairments, disabilities and health related quality of life after treatment for breast cancer: a follow-up study years after surgery. Disabil Rehabil.

[B31] Ripat J, Etcheverry E, Cooper J, Tate R (2001). A comparison of the Canadian occupational performance measure and the health assessment questionnaire. Can J Occup Ther.

[B32] Siegel RL, Miller KD, Jemal A (2018). Cancer statistics. CA Cancer J Clin.

[B33] Tryssenaar J, Jones EJ, Lee D (1999). Occupational performance needs of a shelter population. Can J Occup Ther.

[B34] Vrkljan B, Miller-Polgar J (2001). Meaning of occupational engagement in life-threatening illness: A qualitative pilot project. Can J Occup Ther.

